# Crossbow needle therapy of the Miao ethnic minority group for knee osteoarthritis: study protocol for a randomized controlled trial

**DOI:** 10.1186/s13063-018-2730-4

**Published:** 2018-06-27

**Authors:** Jing Fu, Hong-cai Shang, Li-ying Wang, Chen Zhao, Jin Cui, Yan-ping Wang

**Affiliations:** 1 Guiyang University of Chinese Medicine, Guiyang, 550000 China; 2 Key Laboratory of Chinese Internal Medicine of Ministry of Education and Beijing, Beijing University of Chinese Medicine Affiliated Dongzhimen Hospital, Beijing, 100700 China; 3 Institute of Basic Research In Clinical Medicine, China Academy of Chinese Medical Sciences, Beijing, 100700 China; 4 Tianjin University of Traditional Chinese Medicine, Tianjin, 300193 China

**Keywords:** KOA, Crossbow needle therapy, Acupuncture, Randomized controlled trial

## Abstract

**Background:**

Knee osteoarthritis (KOA) is commonly seen and has a high occurrence in the middle-aged and elderly. It is characterized by the degeneration and secondary bone hyperplasia of the articular cartilage; the pathologic changes are irreversible. Therefore, treatment of KOA is mainly focused on relieving pain, reducing inflammation, improving or restoring joint function, delaying disease progression, and increasing quality of life. Crossbow needle therapy of the Miao ethnic minority group is intended for KOA treatment and has been widely used. Studies of small sample size have seen significant improvement on pain relief, stiffness, and joint function.

**Methods/design:**

The trial is a randomized, multicenter, parallel, non-inferiority study. Three hundred and six patients will be randomly assigned to a crossbow needle group (*n* = 153) and an acupuncture group (*n* = 153). Patients in each group will receive treatment every other day, three times a week, 20 times in total. Follow-up will be conducted 15 days and 30 days after treatment. The primary outcome will be the Western Ontario and McMaster Osteoarthritis Index (WOMAC) score at baseline, the end of treatment, first follow-up, and second follow-up. The secondary outcomes will include Lysholm knee score, Japanese Orthopedic Association (JOA) knee score, visual analogue scale (VAS), and the MOS 36-item short-form health survey (SF-36).

**Discussion:**

The results of the trial will compare the efficacy on KOA between crossbow needle group and acupuncture group and will be expected to make a systematic and objective evaluation of crossbow needle therapy.

**Trial registration:**

ChiCTR, ChiCTR-INR-16008032. Registered on 12 March 2016.

**Electronic supplementary material:**

The online version of this article (10.1186/s13063-018-2730-4) contains supplementary material, which is available to authorized users.

## Background

Knee osteoarthritis (KOA) is a common chronic progressive disease characterized by the degeneration and secondary bone hyperplasia of the knee articular cartilage. It is associated with many factors such as age, body mass index, sex, inflammation, long-term improper exercise, and heredity. Its main signs and symptoms include arthralgia, ankylosis, and functional disorders. Together with obesity, KOA is believed to affect mostly Americans aged 50–84 years [1]. Among people aged under 65 years, more than half have reported three or more years of KOA [2]. In China, KOA morbidity varies among areas, but overall is rising every year. It was estimated that 200,000 patients per year would receive joint replacement [3]. With the aging population, KOA is now a major health risk for middle-aged and elderly people.

Damages on articular cartilage are often irreversible. Neither drug therapy nor surgical treatment can stop the progression of KOA, so treatment of KOA is mainly focused on relieving, reducing inflammation, improving or restoring joint function, delaying disease progression, and increasing quality of life [4]. In western medicine, drug therapy and surgical treatment are the two major interventions of KOA. Drug therapy takes effect very soon, but it always comes with certain side effects and gastrointestinal adverse reaction. Surgical treatment tends to be expensive and is often accompanied by contraindications and complications, limiting its wide application. Back in 1996, KOA was confirmed to be one of the 64 indications of acupuncture therapy in Milan, Italy [5]. Acupuncture is a physical therapy and has no side or toxic effects. Many clinical trials have confirmed its effect on relieving pain and improving joint function in KOA treatment [6–10].

With the support of ethnic minority medicine by the Chinese government, many unique therapies have been discovered and applied because of their remarkable therapeutic effect. Of the 40-odd external therapies in the Miao ethnic minority group, crossbow needle therapy has extremely wide application. It combines needling treatment with transdermal drug administration through the needle and is a broad-spectrum therapy. In Miao medicine, the crossbow needle is traditionally applied to treat cold-bone wind syndrome and cat-head wind syndrome [11–14], while in modern clinical medicine, it is used to treat KOA more often. So far, clinical studies of crossbow needle therapy are all small single-center trials [15–18], which lack clinical evidence and are not enough to make a correct and accurate evaluation.

Thus, we designed this multicenter randomized controlled trial to assess the efficacy of crossbow needle therapy on KOA, in the hope of obtaining replicable evidence that could rank high on the clinical evidence hierarchy, providing further guidance for its development and application.

## Methods/design

### Study design and setting

**Fig. 1:**
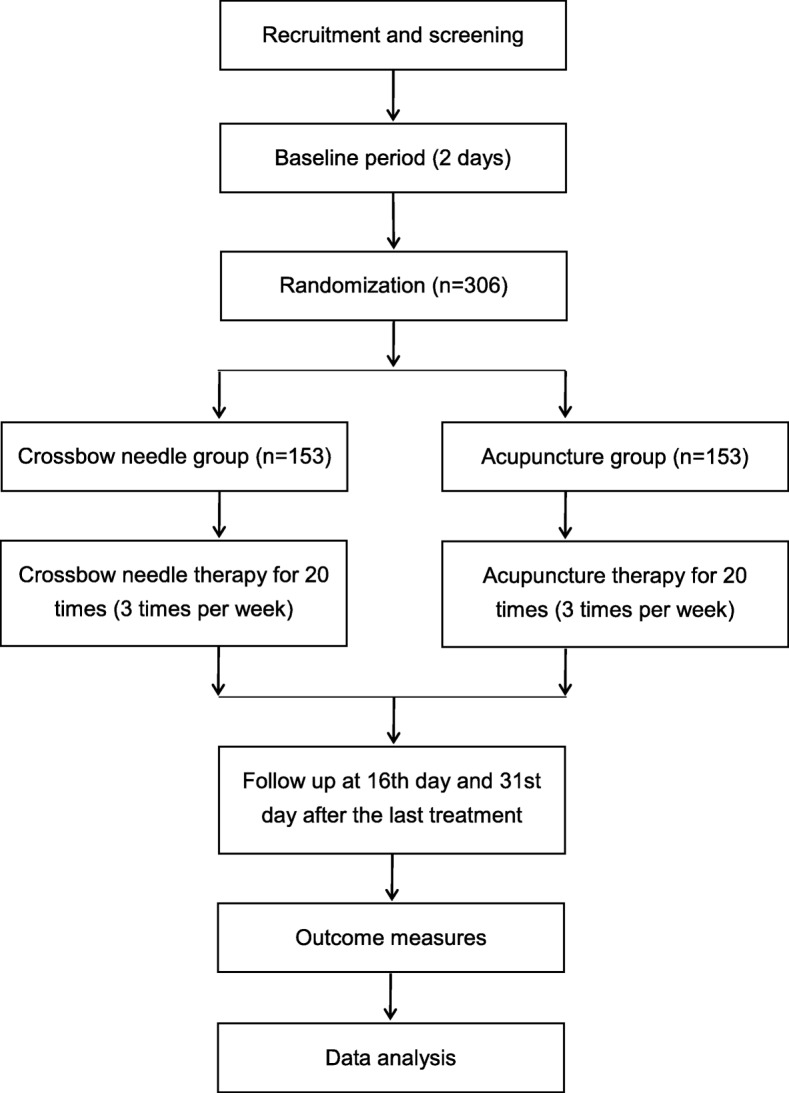
*Flow chart* of the trial

**Fig. 2:**
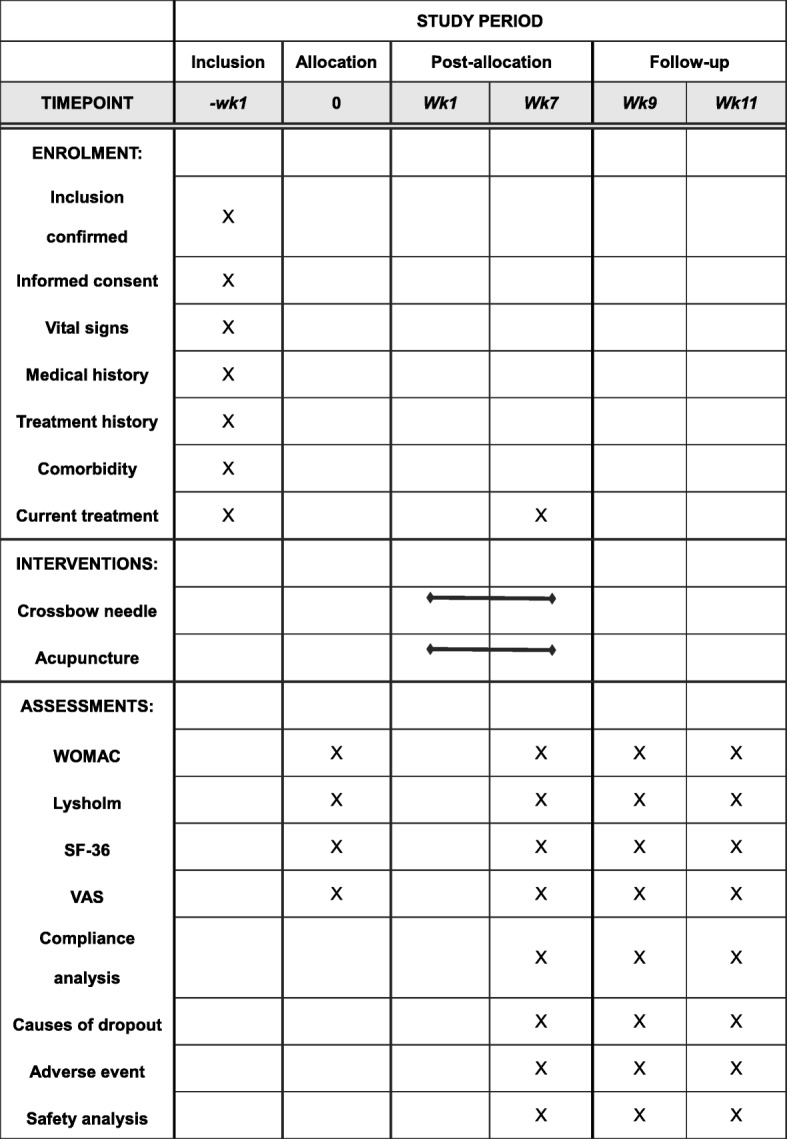
The SPIRIT figure. The schedule of enrollment, interventions, and assessments. WOMAC Western Ontario and McMaster Universities Osteoarthritis Index, SF-36 MOS 36-item short-form health survey, VAS visual analogue scale

This is a parallel, non-inferiority, randomized controlled clinical trial to compare the effect of crossbow needle therapy and regular acupuncture therapy on KOA patients. The patients will be enrolled among outpatients and inpatients from the acupuncture departments of No. 1 Affiliated Hospital of Guiyang University of Chinese Medicine, No. 2 Affiliated Hospital of Guiyang University of Chinese Medicine, and Chinese Medicine Hospital of Qiandongnan Miao and Dong Autonomous Prefecture. Eligible patients will be randomly assigned to a crossbow needle group and an acupuncture group in a 1:1 ratio. All items from the World Health Organization Trial Registration Data Set are shown in Table 1. The study flowchart is provided in Fig. 1. A Standard Protocol Items: Recommendations for Interventional Trials (SPIRIT) checklist and figure are provided respectively in Additional file 1 and Fig. 2.

**Table 1:** All items from the World Health Organization Trial Registration Data Set (SPIRIT checklist, item 2b)

Data category	Information
Primary registry and trial identifying number	http://www.chictr.org.cn; ChiCTR-INR-16008032
Date of registration in primary registry	12 March 2016
Secondary identifying numbers	–
Source(s) of monetary or material support	Special research project of traditional Chinese medicine in 2015 (201507006–01)
Primary sponsor	Institute of Basic Research In Clinical Medicine, China Academy of Chinese Medical Sciences
Secondary sponsor(s)	–
Contact for public queries	Yuexi Wang; wangyue_xi@l26.com
Contact for scientific queries	Yuexi Wang, Institute of Basic Research In Clinical Medicine, China Academy of Chinese Medical Sciences, Beijing 100,700, China.
Public title	Miao medicine in knee osteoarthritis study
Scientific title	Crossbow needle therapy of the Miao ethnic minority group on knee osteoarthritis: study protocol for a multicenter, randomized controlled trial
Countries of recruitment	China
Health condition(s) or problem(s) studied	knee osteoarthritis, pain/ankylosis/joint function disturbance
Intervention(s)	Crossbow needle therapy of the Miao ethnic minority group
Key inclusion and exclusion criteria	*Inclusion criteria* Patients who meet all of the following criteria will be enrolled in the trial: (1) aged 40–75 years; (2) meeting the above diagnostic criteria 1 and 2 (patients graded 1, 2, or 3 osteoarthritis by Kellgren-Lawrecne scale); (3) meeting the above diagnostic criteria 1, 4, 5, and 6; (4) the more affected side to be included when KOA is present on both sides; (5) no intervention performed 1 week before enrollment; (6) aware of all the tasks involved in the trial and prepared to comply with treatment; (7) willingness to sign written informed consent. *Exclusion criteria* Patients who meet any of the following criteria will be excluded from the trial: (1) failed to meet the diagnostic criteria and inclusion criteria; (2) women in pregnancy or lactation; (3) susceptible to allergy; (4) other knee disorders; (5) contusion or sprain in ankle or foot, or other disorders that affect normal walking; (6) ankle/foot deformity or pain; (7) skin disorders or deep swelling at the treated site; (8) other severe primary diseases or complications.
Study type	Multicenter prospective randomized trial
Date of first enrolment	13 April 2016
Target sample size	306
Recruitment status	Recruiting
Primary outcome(s)	The Western Ontario and McMaster Universities Osteoarthritis Index (WOMAC)
Key secondary outcomes	The visual analogue scale (VAS); the Japanese Orthopedic Association (JOA) score; the MOS 36-item short-form health survey (SF-36)

### Study population

#### Diagnostic criteria

The diagnostic criteria [19] are:

1. Frequent knee pain in the recent month;
2. Osteophytes at the joint margin by X-ray;
3. Diagnosis confirmed by joint fluid examination;
4. Aged > 40 years;
5. Morning stiffness < 30 min;
6. Bony crepitus.

Those who meet 1 and 2, or 1, 3, 5, and 6, or 1, 4, 5, and 6 can be diagnosed with KOA. In terms of practical considerations, we will only recruit patients who meet 1 and 2, or 1, 4, 5, and 6.

#### Inclusion criteria

Patients who meet all of the following criteria will be enrolled in the trial:

1. Aged 40–75 years;
2. Meeting the above diagnostic criteria 1 and 2 (patients graded 1, 2, or 3 osteoarthritis by Kellgren-Lawrecne scale [20]);
3. Meeting the above diagnostic criteria 1, 4,5, and 6;
4. The more affected side to be included when KOA is present on both sides;
5. No intervention performed one week before enrollment;
6. Aware of all the tasks involved in the trial and prepared to comply with treatment;
7. Willingness to sign written informed consent.

#### Exclusion criteria

Patients who meet any of the following criteria will be excluded from the trial:

1. Failed to meet the diagnostic criteria and inclusion criteria;
2. Women in pregnancy or lactation;
3. Susceptible to allergy;
4. Other knee disorders;
5. Contusion or sprain in ankle or foot, or other disorders that affect normal walking;
6. Ankle/foot deformity or pain;
7. Skin disorders or deep swelling at the treated site;
8. Other severe primary diseases or complications.

### Sample size

The trial is designed to determine the efficacy of crossbow needle therapy on KOA and prove that its clinical efficacy is not inferior to normal acupuncture therapy; therefore, we chose a non-inferiority trial design [21]. Sample size calculation is based on the formula $n=2{\left[\frac{\left(\mu \alpha +\mu \beta \right)s}{\delta}\right]}^2$, in which μα and μβ are constants and *s* is the combined standard deviation. We set *α* at 0.05 (one-sided), *β* at 0.10, *δ* at 1.5, and *s* at 4.0 (*δ* and *s* based on expert consensus to determine the formula parameters, but not clear as currently), with *μα = μ*_0.05_ = 1.645 and *μβ = μ*_0.10_ = 1.282. After calculation, the sample size of each group should be around 122. Assuming a 20% dropout rate, each group needs at least 153 patients; 306 patients will be recruited in the trial.

### Randomization and blinding

There are two investigators at each center (referred to as A and B). Investigator A is responsible for entering baseline data of patients and perform randomization, but he will not know the grouping details and will not participate in the treatment. Investigator B is responsible for performing treatment according to the grouping. At each visit, A is responsible for collecting data and entering the data in the electronic data capture (EDC) system. The therapist and the data collector are separate. Statisticians are responsible for data processing and are not aware of the grouping until the end of the trial. Central randomization will be conducted by CLINDA Soft Co., Ltd. (Tianjin, China). A random sequence will be generated with SAS 9.2 (SAS Institute, Inc., Cary, NC, USA) by an independent statistician who is not involved in the study; the sequence will be stored in a central randomization system (interactive web response [IWR] system). Patients will be stratified based on the three different centers and randomly assigned to the crossbow needle group and the acupuncture group in a 1:1 ratio. The crossbow needle group will receive crossbow needle therapy. Regular acupuncture therapy will be performed on the acupuncture group.

In this trial, patients will not be blinded due to the uniqueness of the two therapies. Investigators and statisticians will be blinded. Treatment, data collection, and data analysis will be conducted by trained practitioners, data collectors, and statisticians, respectively, all blinded to one another in order to ensure the reliability of the results.

The conditions for unblinding are: (1) infection or other adverse events (AE)—and these patients will be withdrawn from the trial; or (2) the end of the trial and data analysis.

### Interventions

Patients in both groups will receive two sessions (ten times a session) of treatment. The treatment will be performed every other day, three times per week, 20 times in total. The first follow-up will be conducted 15 days after the last treatment, followed by a second follow-up 15 days later. All practitioners involved in the trial are trained and registered professionals. A training program to standardize all manipulations and filling of a case report form (CRF) will be provided before the trial. Investigators will inform all patients about the possible inconveniences from the trial.

### Crossbow needle group

**Fig. 3:**
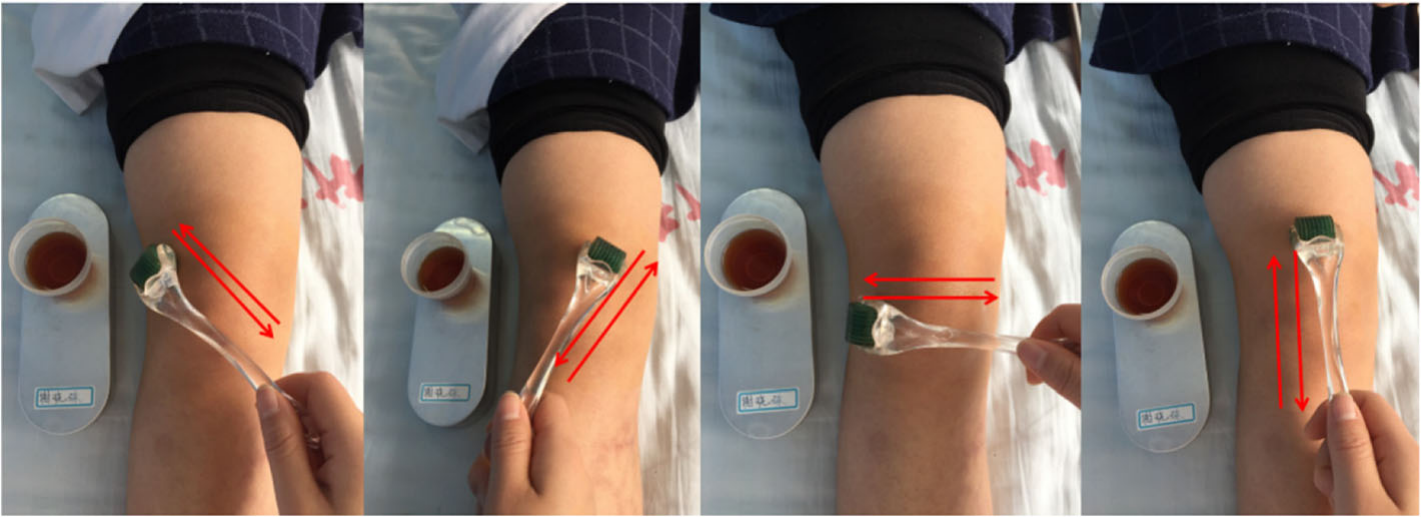
Crossbow needle treatment. The *red arrows* indicate the rolling directions of the skin needle. In the container beside the leg is the herbal liquid

Before treatment, a prescription of herbs containing Sheng Cao Wu (*Radix Aconiti Kusnezoffii*) (20 g), Tou Gu Xiang (*Gaultheria yunnanensis*) (50 g), Hei Gu Teng (*Periploca forrestii*) (30 g), and Ba Jiao Feng (*Alangium chinense*) (15 g) will be ground into a powder, put into a glass container, and soaked in 1000 mL of 50%-proof white liquor for seven days. The obtained clear brown liquid is effective in dispelling wind and eliminating dampness, dispersing numbness and relieving pain, dredging collaterals, and promoting blood circulation [15–18]. Patients in this group will receive crossbow needle therapy. The treatment will use 0.22 × 1.0-mm skin needles (Cloud & Dragon Medical Device Co., Ltd., Wujiang, Zhejiang, China). A practitioner will soak the skin needles in the brown herbal liquid for 10 min before treatment. Patients will be asked to take a supine or sitting position. The soaked skin needles will be applied on the square knee area formed by Xuehai (SP10), Liangqiu (ST34), Yanglingquan (GB34), and Yinlingquan (SP9), with the patella as the center, moving in different directions (Fig. 3). One completion of rolling the skin needle in all directions as displayed in Fig. 3 is seen as one set of application. Skin needles will be dipped into 5 mL of herbal liquid every five sets of application until the liquid is used up. The intensity of application varies from person to person, depending on the tolerance of the patients.

### Acupuncture group

The acupuncture group will be treated with 0.30 × 40 mm and 0.30 × 50 mm disposable sterilized filiform needles (Suzhou Medical Appliance Co., Ltd., Suzhou, Jiangsu, China) applied to Dubi (ST35), Neixiyan (EX-LE4), Xuehai (SP10), Liangqiu (ST34), Yanglingquan (GB34), and Ashi points. The selection of the points is based on the basic theory of acupuncture therapy [22] to treat KOA. Practitioners will perform the treatment according to the national standard of acupuncture manipulation [23–25]. Needles will be inserted and manually manipulated until *de qi* is achieved.

### Combined use of drugs

During the whole period of observation, it is suggested that the patients do not use drugs or other methods for the treatment of KOA in addition to the experimental scheme. During the treatment period, if the patients take medication by themselves or use other methods of treatment due to aggravated symptoms and unbearable knee pain, they will be withdrawn from the trial. The clinicians will make the appropriate treatments according to the specific conditions of the patient until the symptoms are improved.

### Outcome measures

#### Primary outcome measures

We will use the Western Ontario and McMaster Universities Osteoarthritis Index (WOMAC) as the primary outcome measure [26]. The WOMAC is a questionnaire composed of 24 questions to assess physical functional disability. It was developed for patients with osteoarthritis of the hip or knee and measure three subscales: pain; stiffness; and physical function. Higher scores indicate more severe symptoms or physical disability. The Nimodipine method will be applied to calculate the efficacy.

#### Secondary outcome measures

The Lysholm knee score will be used to assess knee function of the patients. The score is rated 0–100, with 25 points attributed to pain, 15 to locking, 10 to swelling, 25 to instability, 10 to stair climbing, and 5 each to squatting, limp, and support.

The Japanese Orthopedic Association (JOA) score is a frequently used scale. It comprises ratings of pain (40 points), range of motion (20 points), gait (20 points), and activities of daily living (20 points). The JOA score will be used to assess the therapeutic effect, with a higher score indicating better effect.

The visual analogue scale (VAS) will be applied to evaluate the pain intensity of the patients. It is a standard tool in pain studies to measure pain intensity. The score is in the range of 0–100 (0 indicating no pain and 100 indicating the worst pain imaginable).

The MOS 36-item short-form health survey (SF-36) will be used to assess the patients’ living standard before and after treatment. The health survey consists of eight dimensions: physical function; role limitations due to physical problems; bodily pain; general health; vitality; social function; role limitations due to mental problems; and mental health. Higher scores represent better quality of life.

### Adherence

Before the start of the trial, the investigators will fully inform the patients of the benefits and the possible inconvenience of the study. The patients will sign the informed consent form and volunteer to participate in the study. Anteroposterior and lateral X-ray of the knee, laboratory examination, and two courses of treatment will be free of charge in order to improve the adherence of the patients. The baseline data of each patient (name, gender, age, address, telephone or contacts of immediate family members) will be recorded in detail, hereby to facilitate the supervision of patients’ adherence. The cases of drop-out, withdrawal, and discontinuation will be recorded and followed up by telephone or even followed up at patients’ homes if necessary.

### Data monitoring and management

Our study group will set up a special data monitoring committee for data management and quality control, which consists of hospitals, company, and institute.

#### Quality control system

All three-level quality monitoring system was established. Clinical research associates (CRAs) were assigned for data monitoring. Level 1 monitoring requires the CRAs in the three hospitals (No. 1 Affiliated Hospital of Guiyang University of Chinese Medicine, No. 2 Affiliated Hospital of Guiyang University of Chinese Medicine, and Chinese Medicine Hospital of Qiandongnan Miao and Dong Autonomous Prefecture) to monitor the quality of the CRFs once a week, fill out the monitoring report, and report to the principal investigator (PI) in time. Level 2 monitoring requires the CRA from CLINDA Soft Co., Ltd. to conduct quality monitoring once every six months, fill out the monitoring report, and report to the project leader of CLINDA in time. Level 3 monitoring requires the person in charge or the technical backbone at the Institute of Basic Research in Clinical Medicine, China Academy of Chinese Medical Sciences to monitor once or twice annually.

#### Data management platform

The data management platform was set up by CLINDA Soft Co., Ltd. It includes the IWR and EDC systems. It includes nine modules, including data entry and statistics. All data will be entered using a double-entry procedure performed by two different individuals.

### Statistical analysis

Data analysis will be conducted in accordance with the intention-to-treat principle. Missing data will be handled using linear mixed effects models. Statisticians and chief investigators will work out an analysis plan and related forms before database lock. The analysis will include case distribution, comparison of basic values, compliance, effectiveness, factors that influence efficacy, and safety. Statistical analysis will be conducted using SAS 9.2 (SAS Institute Inc., Cary, NC, USA). The Wilcoxon rank sum test, *t* test, or variance analysis will be performed for inter-group or intra-group comparison according to data distribution. The Wilcoxon rank sum test will be used to compare ranked data. The chi-square test will be applied to compare enumeration data. All tests will be one-sided. A *p* value < 0.05 will be considered statistically significant.

### Safety monitoring

Possible AEs during crossbow needle treatment include itching, redness, and swelling. Fainting, sticking of needle, hematoma, subcutaneous hematoma, and bleeding might occur during acupuncture treatment. Any AE that occurs during the trial will be recorded in a CRF by researchers and patients will be treated as soon as possible. Patients may choose to withdraw from the trial due to any AE. The sponsor and the data management company CLINDA will monitor the implementation of the trial protocol and filling of CRFs.

## Discussion

Crossbow needle therapy is a broad-spectrum therapy mainly used to treat wind syndromes such as cat-head wind (similar to cold Bi syndrome in traditional Chinese medicine), half-wind (similar to stroke), and tendon-wind (similar to sciatica) [11–14]. In terms of modern clinical medicine, it has been applied in the treatment of KOA, cervical spondylosis, and lumbar disc herniation [16, 27, 28].

Crossbow needle therapy has evolved from ancient times when people of the Miao ethnic minority group killed large animals. The poison they spread on arrows was later applied on human after toxicity attenuation. Prescription of the herbal liquid varies from place to place. Its major components include Sheng Cao Wu (*Radix Aconiti Kusnezoffii*), Bai Long Xu (*Disporum bodinieri*) (Levl. et vaut.) Wang et Tang, Hei Gu Teng (*Periploca forrestii*), Tou Gu Xiang (*Gaultheria yunnanensis*), and Da Xue Teng (*Sargentodoxae caulis*) [29], which have sedative, anti-inflammatory, and analgesic effects, and help promote blood circulation and remove blood stasis [30–32].

**Fig. 4:**
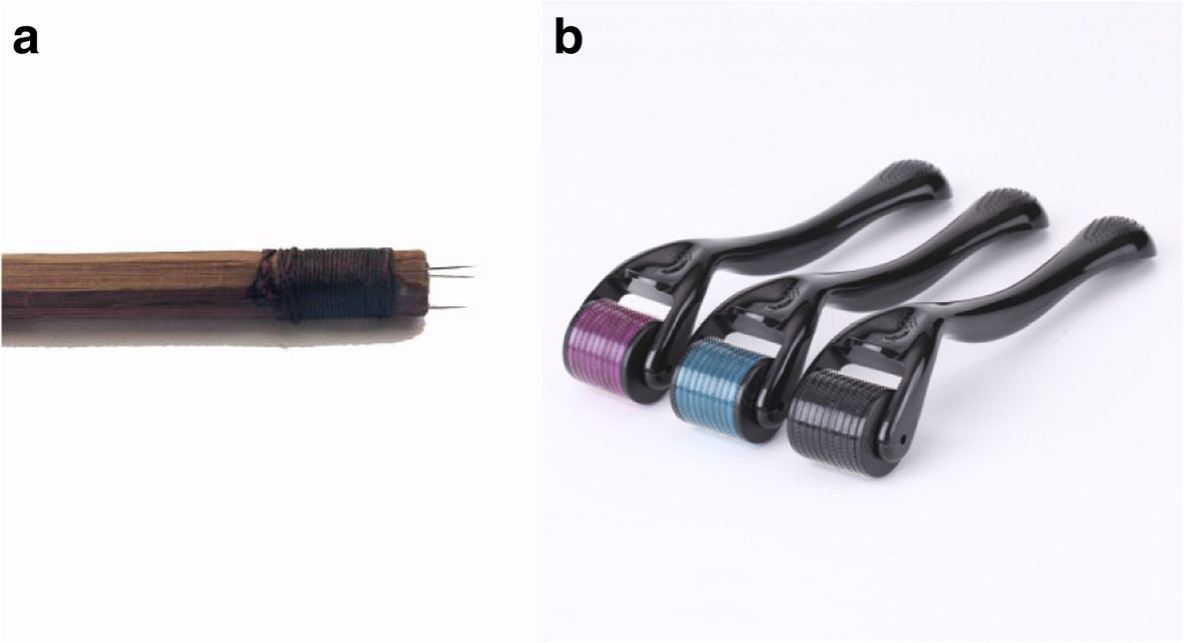
Diagram of ancient crossbow needle and skin needle. **a** An ancient crossbow needle. **b** Skin needles

Ancient crossbow needles were made of sewing needles and bamboo chopsticks or wooden sticks. One or several sewing needles would be stuck into one end of a 15-cm stick, with the needle tip 2 or 3 mm away from the stick (Fig. 4a). Needles would be dipped into the herbal liquid before each needling.

The skin needle is seen as a refined equivalent of the ancient crossbow needle. It is made up of a needle cylinder and a handle, with 30-odd needles embedded in the cylinder. Practitioners usually perform rolling motions along meridians in clinical practice. Compared with the ancient crossbow needle, the skin needle is able to needle more skin area in one application and keep stable contacts with the skin, which reduces painful stimuli. The needle in the cylinder is much thinner than the ancient crossbow needle, decreasing the risk of local hematoma and puncture of internal organs, and easier for young or vulnerable patients to accept skin needle treatment (Fig. 4b) [33, 34].

Administering drugs through the skin is the core of crossbow needle therapy. According to the basic theory of Miao medicine, internal toxins can be driven out of the human body through sweat pores and hair pores on the skin by opening these pores and regulating body temperature. The toxin mentioned here is the source of all diseases in Miao medicine, which believes that all diseases arise from pathogenic toxins. Crossbow needle therapy was developed and intended to open sweat and hair pores by stimulating the skin and administering drugs through the skin into meridians and finally helping drive out internal toxins [35].

According to the basic theory of traditional Chinese medicine, the skin is part of the meridian system and is the external manifestation of the meridian *qi*. On one hand, it is the outermost layer of the human body and plays a defensive role. On the other hand, it connects and affects the inner viscera through the meridian system. When needling is performed, stimuli transmit from the skin through collaterals and meridians to the inner viscera. The process invigorates *Wei qi*, regulates *qi* and blood, balances yin and yang, and finally helps to prevent and treat diseases [36].

From the viewpoint of modern medicine, the skin is the largest organ of the human body and functions to defend the body and regulate immunity. Cuticle layer, pilosebaceous duct, and sweat duct are the three approaches for transdermal drug absorption. The latter two cover only 0.1% of the skin area, so the cuticle layer is the major approach [37]. Nevertheless, the cells in this layer are keratinized dead cells, which can limit the entry of external substances, including drugs. Penetration enhancers enable some small molecule drugs to pass through the skin and reach effective concentrations, but not for most drugs, especially macromolecular drugs [38]. Crossbow needle therapy can solve the problem by puncturing the cuticle layer and passing drugs into the corium layer through the needle puncture holes. This way, drugs can enter the capillary network and reach therapeutic targets at a fast speed with enough concentration, which greatly reduces drug dose and increases drug effect [39, 40]. To date, crossbow needle therapy has been mainly applied on transdermal macromolecular drug delivery [41].

The trial has certain limitations. Drawing knee synovial fluid always leaves a wound, which does not suggest skin needle application should be followed. Therefore, the trial does not include a design to draw knee synovial fluid and conduct further tests. Without these tests, we may lose some objective indexes to evaluate the efficacy of crossbow needle therapy. We will continue to optimize the design in our further study.

### Trial status

The trial is currently recruiting patients and expected to end in May 2018. The first patient was enrolled in April 2016.

## Additional files

Additional file 1: SPIRIT 2013 checklist: recommended items to address in a clinical trial protocol and related documents. (DOC 119 kb)

Additional file 2: Model consent form. (DOC 55 kb)

## Abbreviations

CRF: Case report form
EDC: Electronic data capture
IWR: Interactive web response
JOA: Japanese Orthopedic Association
KOA: Knee osteoarthritis
PI: Principal investigator
SF-36: MOS 36-item short-form health survey
SPIRIT: Standard Protocol Items: Recommendations for Interventional Trials
VAS: Visual analogue scale
WOMAC: Western Ontario and McMaster Universities Osteoarthritis Index
