# Supplementary material for: Crossbow needle therapy of the Miao ethnic minority group for knee osteoarthritis: study protocol for a randomized controlled trial
Source: Trials. 2018 Jun 27;19:338. doi: 10.1186/s13063-018-2730-4 (PMC6020384; doi:10.1186/s13063-018-2730-4)
Supplement: Supplementary file 2 — Model consent form. (DOC 55 kb) [file 13063_2018_2730_MOESM2_ESM.doc]

**Informed consent·Informed notification**

Dear patient:

You have been diagnosed as Knee osteoarthritis. We invite you to participate in a study. This study is Special research project of traditional Chinese medicine in 2015 and the project number is: 201507006-01. This study protocol was reviewed and approved by the ethics committee of No.1 Affiliated Hospital of Guiyang University of Chinese Medicine.

Before you make your decision to participate in this study, please read the following as carefully as possible. It helps you to understand the details and objective, procedure and duration of the study, as well as the benefits, risks and discomfort that may be brought to you after taking part in the study. You can also discuss with your relatives and friends, or consult the doctors to help you make a decision.

1. **Background and objective**

**1.1 Burden and treatment status of the disease**

Knee osteoarthritis (KOA), also known as degeneration of knee joint or hyperosteogeny of knee joint, which is commonly found in middle-aged and elder people. It is a common chronic progressive disease, with the degeneration of articular cartilage and secondary hyperosteogeny as the main pathological changes. The onset is related to many factors, such as age, body mass index, gender, inflammation, long-term irrational exercise and heredity, etc. Its manifestations include joint swelling, stiffness and dysfunction, and it is called “undead cancer”. KOA and obesity are considered to be the most common chronic diseases affecting the health of 50-84 years old American. More than half of the people under 65 years old have symptoms of KOA, and their symptoms persist for more than 3 years. In China, the prevalence varies from place to place, but it is increasing year by year. The annual conservative estimate of patients receiving joint replacement in China is about 200,000. With the aging of population, the prevalence rate will continue to increase. Hence, the disease has indeed become a major threat to the health of the middle-aged and elder people.

The destruction of articular cartilage is often irreversible. The existing medical measures, whether medication, surgery or physical therapy, cannot change the pathological trend of destruction of articular cartilage. Therefore, the purpose of KOA treatment is mainly to relieve or eliminate pain, reduce inflammation, improve or restore joint function, delay disease progression and improve the quality of life of patients. For this purpose, western medicine mainly adopts drug therapy and surgery. Medicine has quick effect, but has some side effects, where a long-term use may lead to risk of gastrointestinal adverse events. Meanwhile, surgical treatment is expensive, and may have many contraindications and complications, which greatly limits its popularization and application. Knee pain is a chronic pain in the motor system. As early as in 1996, it was recognized as one of the 64 indications of acupuncture in Italy Milan conference. In addition, acupuncture is a physical therapy and has no toxic side effects, which has been confirmed by a large number of clinical studies to be able to significantly relieve pain and improve joint function in patients with KOA, and has been accepted by scholars in different countries as well as the majority of patients.

In recent years, with the vigorous support from the government and the development of ethnic medicine, many unique treatment methods in traditional ethnomedicine have been gradually applied due to their remarkable therapeutic effects. The crossbow needle therapy is one of the widely used more than 40 kinds of external therapeutic methods in Miao medicine. It combines the effects of acupuncture and medication, and belongs to a broad-spectrum treatment method. Miao medicine is mainly used for the treatment of diseases such as “Leng Gu Feng” and “Mao Tou Feng”, etc., and is often used for the treatment of KOA in modern clinical studies. However, previous clinical studies were single-center studies with a small sample size, with insufficient clinical evidence grade and lacking of reliability, and thus were unable to make accurate and fair evaluation of the therapeutic effect of crossbow needle therapy in Miao medicine for the treatment of KOA, and their results had a low repeatability and representiveness.

**1.2 Objective of the study**

This multicenter randomized controlled trial aims to evaluate the efficacy of crossbow needle therapy in Miao medicine on KOA, so as to obtain clinical evidence with a high grade, repeatability and representiveness, to further guide the clinical popularization and application, and thus to develop and heritage the Miao medicine theory as well as improve the comprehensive service ability of Miao medicine.

**1.3 Participating units and expected number of subjects**

The participating units include the Institute of Basic Research In Clinical Medicine, China Academy of Chinese Medical Sciences; No.1 Affiliated Hospital of Guiyang University of Chinese Medicine; No.2 Affiliated Hospital of Guiyang University of Chinese Medicine; and Chinese Medicine Hospital of Qiandongnan Miao and Dong Autonomous Prefecture. We expect to enroll 306 subjects.

**2. Which subjects can’t participate in the study?**

Patients who meet any of the following criteria will be excluded from the trial:

(1) Failed to meet the diagnostic criteria and inclusion criteria

(2) Women in pregnancy or lactation

(3) Susceptible to allergy

(4) Other knee disorders

(5) Contusion or sprain in ankle or foot, or other disorders that affect normal walking

(6) Ankle/foot deformity or pain

(7) Skin disorders or deep swelling at the treated site

(8) Other severe primary diseases or complications

**3. What will you need to do if you participate in the study?**

**3.1** Before you are enrolled in the study, you will be asked to undergo the following examination for confirming eligibility: the doctors will ask and record your general information, including gender, age, height, body weight, living environment, education level, medical history and so on, and perform relevant physical examinations.

You will be asked to undergo examinations such as blood routine, liver function, kidney function, knee joint X-ray.

**3.2** If the above screening results are in conformity with the inclusion criteria, the study will be performed according to following steps:

(1) This is a multicenter randomized controlled trial. If you are eligible and willing to participate in the study, the doctor will inform you that you will be given crossbow needle therapy or acupuncture according to the results of random grouping provided by computer software. You have 1/2 possibility to be assigned to either of these two different groups, and neither you nor your doctor can know and choose the treatment in advance. Subjects in both groups will be treated 3 times a week, 10 times for a course of treatment, a total of 2 courses. And you will need to cooperate with 2 times of follow-up which will be conducted at half a month (15±1 days) and one month (30±1 days) after the end of treatment.

(2) The crossbow needles used in this study are the skin needles produced by Wujiang Cloud & Dragon Medical Device Co., Ltd. (producer license: Suzhou Food and Drug Administration to the production of Xu 2001-0046; Registration certificate number: Suzhou mechanical note 20142270173; Specification: 0.22×1.0 mm (diameter of needle×long diameter of needle).

The crossbow regimen was derived from the empirical prescription of Miao ethic minority group, and the crossbow liquor was prepared by our study group according to folk brewing method in Miao medicine.

The acupuncture needles are the Huatuo disposable acupuncture needles produced by Suzhou Medical Appliance Co., Ltd. (producer license: Suzhou Food and Drug Administration to the production of Xu 2001 0020; Registration certificate number: Appliance Permit Number 2270864 of 2012 by Suzhou Food and Drug Administration; Specification: φ0.30×40 mm, φ0.30×50 mm).

(3) You need to cooperate with filling out the following scales before treatment, at the end of treatment and at 2 times of follow-up: the Western Ontario and McMaster Osteoarthritis Index (WOMAC) score, Lysholm knee score, visual analogue scale (VAS), the MOS 36-item short-form health survey (SF-36) and Japanese Orthopaedic Association (JOA) score.

**3.3** Other items you need to cooperate with

(1) You must follow the doctor’s schedule to visit the hospital and follow-up. The follow-up is very important, because the doctor will determine whether the treatment is really effective. At the same time, you are also responsible for reporting any change in your physical and mental aspects to the doctor, whether you believe this change is related to this study or not.

(2) During the study period, you can't use drugs and methods that interfere with KOA except for this study protocol. If you need other treatment, please contact your doctor beforehand.

**4. Possible benefits of participating in the study**

You may benefit from this study, which includes the possibility of improvement in your condition. During this trial, only the knee joint on one side with severe condition will be treated. If both of your knees suffer from osteoarthritis, we can provide you with up to 5 times of free symptomatic treatment for the knee joint after the end of the trial (acupuncture/crossbow medicine needle). The results of this project may eventually be applied and popularized in clinical practice.

**5. Possible adverse events, risks, discomfort and inconvenience**

There may be adverse events in the treatment with crossbow needle and acupuncture, which are uncommon and slight. During the acupuncture treatment process, you may have sour, numb, heavy and swelling sensation, which are normal reactions of acupuncture, or you may appear syncope due to your body constitution or nervousness, which can be alleviated by stopping acupuncture and having appropriate rest. There may be pruritus, slight pain and other phenomena after the treatment with crossbow needle therapy and acupuncture, which can disappear after receiving simple treatment. If there is infection at the treatment site, your doctor will treat it in time until symptoms disappear.

If there is any discomfort during the study period, or any new changes in the conditions, or any unexpected situations, whether it is related to the crossbow needle and acupuncture treatment or not, you should inform your doctor in time, and he/she will make a judgement about this and provide proper medical treatment.

You need to go to the hospital on time for follow up and some examinations, which may cause trouble or inconvenience to you.

**6. Related expenses**

Doctors will do their best to prevent and treat the dangers that may be caused by this study. If there are adverse events in clinical trial, medical experts committee will identify whether it is related with crossbow needle treatment and acupuncture or trial process, and the sponsor will provide the cost of treatment and make the corresponding economic compensation for the damage related to study drug or trial process according to the description in *Good Clinical Practice*.

We will undertake the expenses related to each special examination and assessment (blood routine, liver and kidney function, knee joint X-ray) and the 20 times of symptomatic treatment for your knee joint.

**7. Is personal information confidential?**

Your medical records (study records/CRF, laboratory tests, etc.) will be kept in your hospital. The doctor will record the test results in your medical record. The investigators, the ethics committee, and the drug supervision and management department will be allowed to look up your medical records. Any public report on the results of this study will not reveal your personal identity. We will make every effort to protect the privacy of your personal medical data within the scope of the law.

**8. How to obtain more information?**

You can put forward any question about this study at any time, and can get the answers accordingly.

If there is any important new information in the course of the study that may affect your continued participation in the research, your doctor will inform you in time.

**9. Choose to participate in and withdraw from the study in the midway voluntarily**

Whether to participate in the study is entirely dependent on your wishes. You may refuse to take part in the study or withdraw from this study at any time during the trial process, which will not affect the relationship between you and your doctor, and will not affect your medical or other losses.

For your best interest, a doctor or investigator may terminate your participation at any time during the study.

If you withdraw from the study for any reason, you may be asked about the situation since you receive the crossbow needle and acupuncture treatment. If the doctor thinks it is necessary, you may also be required for laboratory examination and physical examination.

**10. What to do now?**

Whether to participate in this study is decided by yourself (and your family).

Before you make any decision to participate in the study, please consult your doctor as much as possible.

Thank you for reading the above materials. If you decide to take part in this study, please tell your doctor and he/she will arrange all the related matters for you.

Please keep this copy of the informed consent.

**11. Ethics office telephone/fax: 0851-85639710.**

**Informed consent·Informed signature**

**Name of clinical study project:** A clinical study on crossbow needle therapy of Miao medicine for the treatment of knee osteoarthritis

**Project undertaking unit:** Guiyang University of Chinese Medicine

**Project cooperating unit:** No.1 Affiliated Hospital of Guiyang University of Chinese Medicine; No.2 Affiliated Hospital of Guiyang University of Chinese Medicine; Chinese Medicine Hospital of Qiandongnan Miao and Dong Autonomous Prefecture

**Project number:** 201507006-01

**Declaration**

I have read the above introduction to this study and have the opportunity to discuss and raise questions with doctors in this study. All the questions I put forward have been answered satisfactorily.

I understand the possible risks and benefits of participating in this study. I know that participating in the study is voluntary, and I confirm that I have enough time to think about it and understand that:

- I can ask the doctor for more information at any time.
- I can withdraw from this study at any time without discrimination or retaliation, and my medical treatment and rights will not be affected.

I also know that when I drop out of the study, especially because of drug reasons, if I tell the doctor about the change of my condition and complete the physical examination and physio-chemical examination, it will be very beneficial for the whole study.

If I need to take any other medical treatment because of the change of condition, I will ask the doctor in advance, or tell the doctor honestly after the event.

I agree with the ethics committee of the drug supervision department or the representative of the sponsor to look up my study data.

I will get a copy of the informed consent that is signed and dated.

Finally, I decide to agree to participate in this study and to ensure that I will try to comply with the doctor's orders as much as possible.

Signature of patient: Date:

Contact phone number:

I confirm that the details of the trial have been explained to the patient, including his/her rights as well as possible benefits and risks, and a copy of the informed consent with signature has been given to the patient.

Signature of doctor: 　 Date:

Work phone number:
